# Associations between Interleukin-1 Gene Polymorphisms and Coronary Heart Disease Risk: A Meta-Analysis

**DOI:** 10.1371/journal.pone.0045641

**Published:** 2012-09-19

**Authors:** Liang Zhou, Jianguang Cai, Gang Liu, Yuan Wei, Hui Tang

**Affiliations:** 1 Department of Sports Physiology, Hunan University of Science and Technology, Xiangtan, Hunan, China; 2 Department of Sports Physiology, Guangzhou Sports University, Guangzhou, Guangdong, China; University of Freiburg, Germany

## Abstract

**Objective:**

A great number of studies regarding the associations between *IL-1B-511*, *IL-1B+3954* and *IL-1RN VNTR* polymorphisms within the *IL-1*gene cluster and coronary heart disease (CHD) have been published. However, results have been inconsistent. In this study, a meta-analysis was performed to investigate the associations.

**Methods:**

Published literature from PubMed and Embase databases were searched for eligible publications. Pooled odds ratios (ORs) with 95% confidence intervals (CIs) were calculated using random- or fixed- effect model.

**Results:**

Thirteen studies (3,219 cases/2,445 controls) for *IL-1B-511* polymorphism, nine studies (1,828 cases/1,818 controls) for *IL-1B+3954* polymorphism and twelve studies (2,987 cases/ 2,208 controls) for *IL-1RN VNTR* polymorphism were included in this meta analysis. The results indicated that both *IL-1B-511* and *IL-1B+3954* polymorphisms were not associated with CHD risk (*IL-1B-511* T vs. C: OR = 0.98, 95%CI 0.87–1.09; *IL-1B+3954* T vs. C: OR = 1.06, 95%CI 0.95–1.19). Similarly, there was no association between *IL-1RN VNTR* polymorphism and CHD risk (*2 vs. L: OR = 1.00, 95%CI 0.85–1.17).

**Conclusions:**

This meta-analysis suggested that there were no associations between *IL-1* gene cluster polymorphisms and CHD.

## Introduction

Coronary artery disease (CHD) is the most common form of cardiovascular disease which remains the leading cause of mortality and morbidity worldwide [1]. CHD accounts for 7.3 million death in 2008 [2]. CHD is an extremely complex and multifactorial disease, which is attributed to multiple genetic and environment factors and their interactions.

It is well known that atherosclerosis is the underlying pathology of CHD through a slowly progressing lesion formation and luminal narrowing of arteries [3]. Growing evidence has suggested that inflammation plays an important role in the initiation and progression of atherosclerosis, which is recognized as a progressive inflammatory disorder [3]–[5]. The adventitial inflammation of advanced plaques can be attributed to the release of some enzymes, cytokines and chemokines [3], [6]. Interleukin-1 (IL-1) family is a critical mediator of inflammatory response with two agonists (IL-1α and IL-1β) and one antagonist (Interleukin-1 Receptor antagonist: IL-1Ra) [7]. The *IL-1* gene cluster, located within 430 kb region on chromosome 2 (2q13-21), contains *IL-1A*, *IL-1B* and *IL-1RN* (encoding IL-1α, IL-1β and IL-1Ra, respectively) genes [8]. Three single nucleotide polymorphisms (SNPs) of the *IL-1* gene cluster have been most frequently studied in relation to CHD risk: one SNP at promoter position −511 C/T and another one in exon 5 at position +3954 C/T of the *IL-1B* gene and a variable number of tandem repeats (VNTR) of 86 bp polymorphism in intron 2 of *IL-1RN* gene [9], [10], which generates a short allele with two repeats (IL-1RN*2) and long alleles with three to six repeats (*IL-1RN L*) [10].

To date, a great number of studies regarding the associations between *IL-1* gene cluster polymorphisms and CHD risk have been published. However, results have been inconsistent [11]–[29]. Chen et al performed a meta-analysis in Chinese to investigate the associations between *IL-1* gene polymorphisms and CHD in 2000 [30] and only seven papers were included in that meta-analysis. In addition, it did not investigate the relationship between *IL-1B+3954* polymorphism and CHD risk. Therefore, in this study, we performed a meta-analysis to further clarify the associations between *IL-1B-511*, *IL-1B+3954* and *IL-1RN VNTR* polymorphisms and CHD risk.

## Materials and Methods

### Literature and search strategy

The PubMed and Embase database searches were performed to identify all eligible articles. The search strategy involved the use of combination of the following key words: (Interleukin-1 or IL-1) and (variant or variation or polymorphism) and (coronary disease or coronary heart disease or coronary artery disease or myocardial infarct or ischemic heart disease or CHD or IHD or MI or cardiovascular disease or heart disease OR angina). The publication languages were restricted to English and Chinese. The reference lists of retrieved articles were hand-searched. If more than one article was published using the same study data, only the study with the largest sample size was included. The literature search was updated on April 20, 2012.

### Inclusion criteria and data extraction

Studies were included in the analysis if they met the following inclusion criteria: (1) a case-control or cohort study; (2) evaluating the associations of IL-1 genetic polymorphisms (include *IL-1B-511*, *IL-1B+3954* or *IL-1RN VNTR*) with CHD risk; and (3) providing sufficient data for calculation of an odds ratio (OR) with 95% confidence interval (CI). The following information was extracted from each study: (1) name of first author; (2) year of publication; (3) country of origin; (4) ethnicity of the study population; (5) source of controls (population- or hospital-based); (6) sample size of cases and controls; (7) cardiovascular end point; (8) gender distribution and mean age of subjects in cases and controls; (9) genotype distributions in cases and controls; and (10) *p* value for the test of Hardy–Weinberg equilibrium (HWE) in controls. Two authors independently assessed the articles for compliance with the inclusion criteria, and disagreement was followed by discussion until consensus was reached.

### Statistical analysis

The associations between *IL-1* genetic polymorphisms and CHD risk were estimated by calculating pooled ORs and 95%CI under multiplicative, co-dominant, dominant, and recessive genetic models, respectively. The significance of pooled ORs was determined by *Z* tests (*p*<0.05 was considered statistically significant). A Q test was performed to evaluate whether the heterogeneity existed. A random- (DerSimonian-Laird method) [31] or fixed- (Mantel-Haenszel method) [32] effects model was used to calculate the pooled ORs in the presence (*p*< = 0.10) or absence (*p*>0.10) of heterogeneity. Meta-regression was performed to explore the potentially important sources of between-study heterogeneity. Subgroup analyses based on ethnicity, cardiovascular end point, source of controls and sample size (n<400 vs.n≥400) were also performed. Sensitivity analysis, removing one study at a time, was performed to evaluate the stability of the results. Begg's funnel plot, a scatter plot of effect against study size, was generated as a visual aid to detect bias or systematic heterogeneity [33]. Publication bias was assessed by Egger's test [34] (*p*<0.05 was considered statistically significant). Data analysis was performed using STATA version 11 (StataCorp LP, College Station, TX, USA).

## Results

### Characteristics of the studies

**Figure 1:**
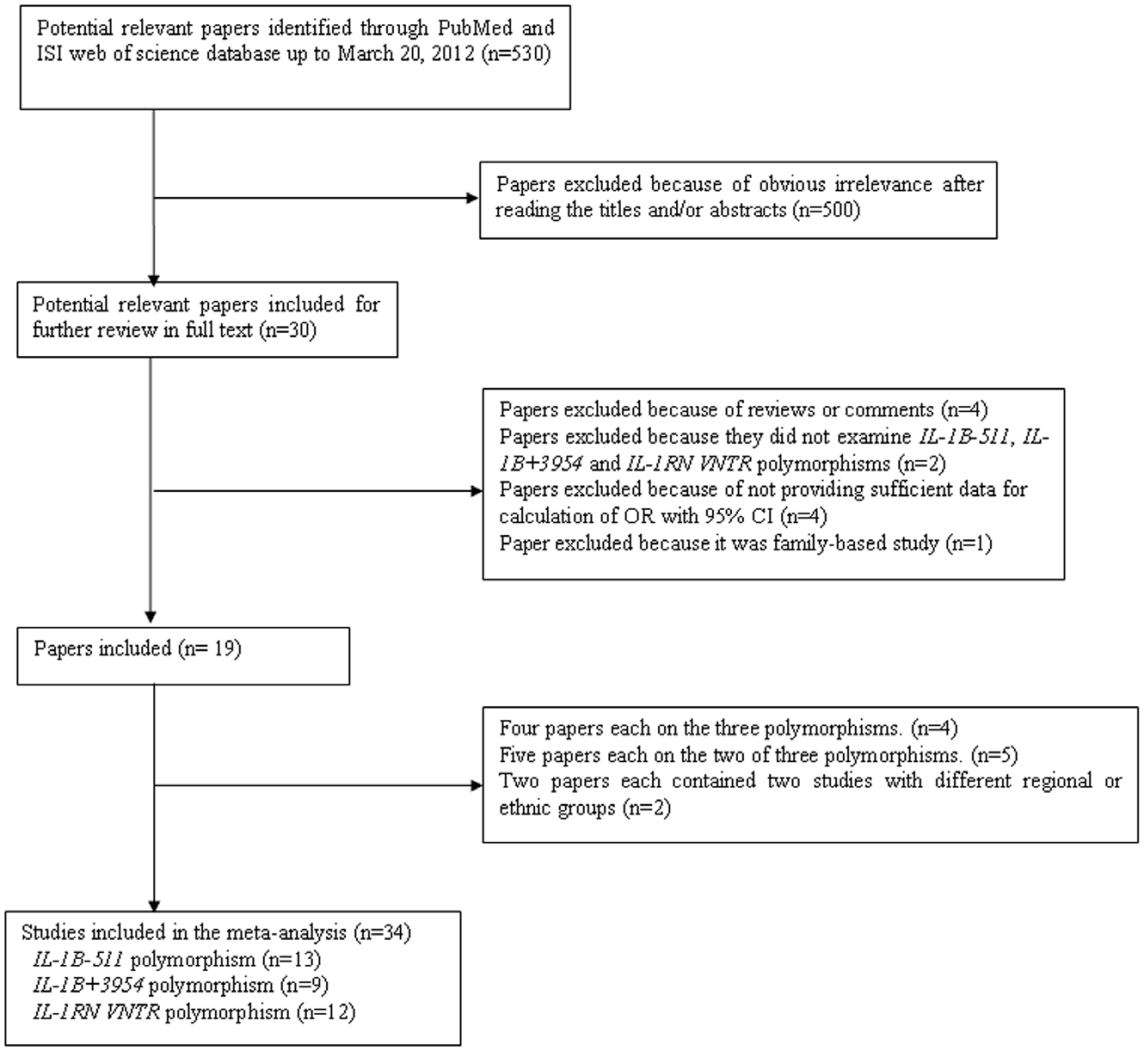
Selection of articles for inclusion in meta-analysis.

A total of 530 potentially relevant papers were identified based on the search strategy. Of these, 500 papers were excluded because of obvious irrelevance by reading their titles and abstracts. After the full texts were read, four papers were excluded because they didn't provide sufficient data for calculation of OR with 95% CI [35]–[38]; two papers were excluded because of examining the associations of other genetic polymorphisms rather than the three polymorphisms studied in our analysis [39]–[40]; another paper was excluded because it was family-based study [41]. In addition, two reviews [30], [42] and two comments [43], [44] were excluded with the exception of the one by Iacoviello et al [12], which provided a new study concerning the association of *IL-1RN VNTR* polymorphism and CHD. Furthermore, more than one study were included in each of the two papers by Francis et al [11] and Rios et al [26], respectively, and they were considered as separate studies in the following data analysis. Thus, thirteen studies [11], [14]–[17], [19]–[22], [26], [27] on *IL-1B-511* polymorphism, nine studies [16], [19]–[24], [27], [29] on *IL-1B+3954* polymorphism, twelve studies [11]–[14], [18]–[21], [25], [27], [28] on *IL-1RN VNTR* polymorphism were included in the final meta-analyses. A flow chart demonstrating the inclusion/exclusion of studies was displayed as Figure 1. The characteristics of the included studies were listed in Table 1.

**Table 1:** Characteristics of studies included in the meta-analysis.

Study	Year	Country	Ethnicity	Source of controls	Sample size (case/control)	End point	Mean age in case/control, (years)	% of male (case/control)	Genotype distribution							*P*_HWE_ a
									Case				Control			
									11	12	22		11	12	22	
*IL-1B-511* polymorphism																
Francis^11^	1999	UK	Caucasian	PB	425/130	CHD	58.9/58.9	77.6/43	574^b^		276^c^		193^b^		67^c^	≥0.05
Francis^11^	1999	UK	Caucasian	PB	248/102	CHD	61.1/57.4	76.5/36	335^b^		161^c^		138^b^		66^c^	≥0.05
Vohnout^14^	2003	Slovakia	Caucasian	HB	335/205	CHD	59.5/56.6	83.6/46.8	151	152	32		90	89	26	0.587
Licastro^15^	2004	Italy	Caucasian	PB	139/198	MI	65/57	100/100	65	60	14		46	65	11	0.075
Iacoviello^16^	2005	Italy	Caucasian	PB	406/419	MI	41/40	85/85	195	180	31		174	187	58	0.495
Zhang^17^	2006	China	Asian	PB	127/152	CHD	52/44	66.9/64.5	25	79	23		38	92	22	0.006
Arman^19^	2008	Turkey	Caucasian	HB	257/170	CHD	58.0/51.4	72.4/53.5	75	130	52		51	74	45	0.094
Geismar^20^	2008	Denmark	Caucasian	HB	96/123	CHD	63.8/61.8	69.1/62.5	43	42	11		60	50	13	0.595
Soylu^21^	2008	Turkey	Caucasian	HB	264/117	ACS	58.3/52.4	73.1/48.7	81	128	55		28	58	31	0.932
Zee^22^	2008	USA	Caucasian	PB	341/341	MI	60.2/60.1	100/100	148	154	39		164	137	40	0.172
Rios^26^	2010	Brazil	African	HB	138/115	CHD	55.7/51.8	64.5/43.5	42	64	32		19	67	29	0.062
Rios^26^	2010	Brazil	Caucasian	HB	276/138	CHD	55.7/53.0	66.7/45.6	80	130	66		47	69	22	0.69
Coker^27^	2011	Turkey	Caucasian	HB	167/235	MI	53.4/53.9	70/43	59	72	36		77	113	45	0.758
*IL-1B+3954* polymorphism																
Iacoviello^16^	2005	Italy	Caucasian	PB	406/419	MI	<50	NA	244	140	14		258	130	14	0.63
Arman^19^	2008	Turkey	Caucasian	HB	257/170	CHD	58.0/51.4	72.4/53.5	151	91	15		93	68	9	0.446
Geismar^20^	2008	Denmark	Caucasian	HB	96/123	CHD	63.8/61.8	69.1/62.5	51	38	7		67	45	11	0.393
Soylu^21^	2008	Turkey	Caucasian	HB	264/117	CHD	58.3/52.4	73.1/48.7	157	93	14		69	41	7	0.783
Zee^22^	2008	USA	Caucasian	PB	341/341	MI	60.2/60.1	100/100	188	130	23		198	123	20	0.877
Stein^23^	2009	Germany	Caucasian	HB	54/50	AMI	50.8/51.7	92.6/94	48^b^		24^c^		48^b^		21^c^	≥0.05
Zhu^24^	2009	China	Asian	HB	100/144	CHD	61.5/60.3	67/67.4	97	3	0		142	2	0	0.933
Coker^27^	2011	Turkey	Caucasian	HB	167/235	MI	53.4/53.9	70/43	86	68	13		136	84	15	0.677
Zeybek^29^	2011	Turkey	Caucasian	PB	143/213	MI	58.9/56.4	68.5/39.9	79	46	18		140	54	19	<0.001
*IL-1RN VNTR* polymorphism																
Francis^11^	1999	UK	Caucasian	PB	425/130	CHD	58.9/58.9	77.6/43	628^b^		222^c^		201^b^		59^c^	≥0.05
Francis^11^	1999	UK	Caucasian	PB	248/102	CHD	61.1/57.4	76.5/36	356^b^		140^c^		171^b^		33^c^	≥0.05
Iacoviello^12^	2000	Italy	Caucasian	PB	158/153	AMI	<50	81.6/	237^b^		79^c^		229^b^		77^c^	≥0.05
Zee^13^	2001	USA	Caucasian	PB	385/385	MI	59.6/59.5	M	219	140	26		218	137	30	0.199
Vohnout^14^	2003	Slovakia	Caucasian	HB	335/205	CHD	59.5/56.6	83.6/46.8	200	114	21		127	68	20	0.02
Kariz^18^	2007	Slovenia	Caucasian	PB	151/223	MI	59.2/66.5	65.6/45.7	87	49	15		134	75	14	0.428
Arman^19^	2008	Turkey	Caucasian	HB	257/170	CHD	58.0/51.4	72.4/53.5	150	84	23		105	56	9	0.67
Geismar^20^	2008	Denmark	Caucasian	HB	96/123	CHD	63.8/61.8	69.1/62.5	59	32	4		68	40	15	0.027
Soylu^21^	2008	Turkey	Caucasian	HB	264/117	CHD	58.3/52.4	73.1/48.7	149	96	19		59	45	13	0.33
Fragoso^25^	2010	Mexico	Mixed	PB	300/248	ACS	59/56	79/79	177	90	33		113	97	38	0.029
Coker^27^	2011	Turkey	Caucasian	HB	167/235	MI	53.4/53.9	70/43	88	52	27		124	77	34	<0.001
Goracy^28^	2011	Poland	Caucasian	HB	201/117	CHD	57.3/55.2	82.1/59.0	93	86	22		57	48	12	0.689

*Notes* For *IL-1B-511* and *IL-1B+3954* polymorphisms, 11 = CC, 12 = CT, 22 = TT; for *IL-1RN VNTR* polymorphism, 11 = L/L, 12 = *2/L, 22 = *2/*2;

PB, Population-based; HB, Hospital-based; NA, not available;

a , *p* value for Hardy-Weinberg equilibrium in controls; ^b,^ allele represented by 1; ^c,^ allele represented by 2;

CHD = coronary artery disease; ACS = acute coronary syndrome; MI = myocardial infarction.

### Quantitative data synthesis

**Figure 2:**
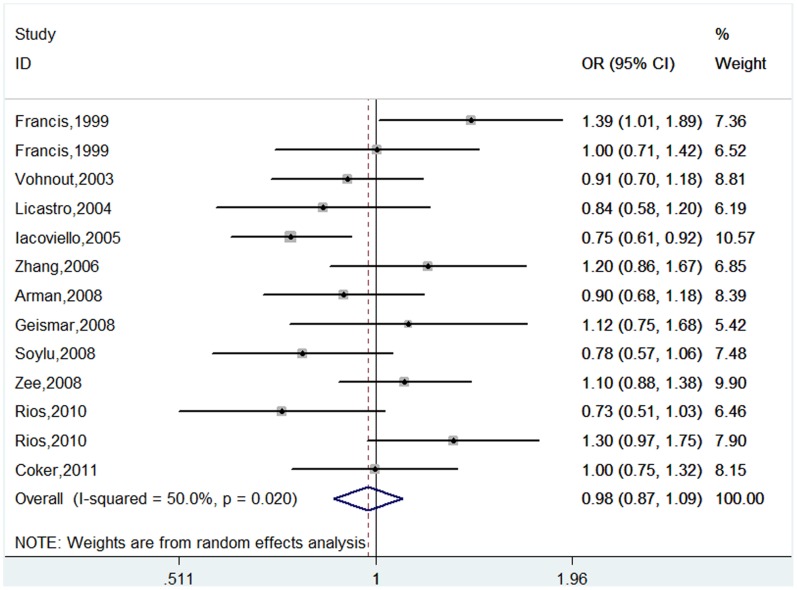
Meta-analysis of the association between *IL-1B -511* polymorphism and CHD risk (T vs. C).

For *IL-1B-511* polymorphism, a total of 3,219 cases and 2,445 controls were identified. Overall, the results showed no significant association between this polymorphism and CHD risk (T vs. C: OR = 0.98, 95%CI 0.87–1.09; TT vs. CC : OR = 0.87, 95%CI 0.67–1.13; TC vs. CC: OR = 0.96, 95%CI 0.84–1.09; TT+TC vs. CC: OR = 0.93, 95%CI 0.79–1.10; TT vs. TC+CC: OR = 0.91, 95%CI 0.74–1.12) (Table 2, Figure 2). In the subgroup analysis, there was no statistically significant association in each subgroup by ethnicity, cardiovascular end point, source of controls and sample size under all genetic models except for the association for Africans under co-dominant and dominant models (Table 2). However, the positive result of subgroup analysis by ethnicity was not reliable for Africans because only one study was performed in African patients.

**Table 2:** Summary ORs and 95%CIs of the association between *IL-1B -511* polymorphism and CHD risk.

Contrasts	No. of studies	T vs. C			No. of studies	TT vs. CC			TC vs. CC			TT+TC vs. CC			TT vs. TC+CC		
		OR	95% CI	*P*_H_		OR	95% CI	*P*_H_	OR	95% CI	*P*_H_	OR	95% CI	*P*_H_	OR	95% CI	*P*_H_
All	13	0.98	0.87–1.09	0.020	11	0.87	0.67–1.13	0.039	0.96	0.84–1.09	0.125	0.93	0.79–1.10	0.069	0.91	0.74–1.12	0.100
Studies in HWE	12	0.96	0.85–1.08	0.022	10	0.83	0.64–1.08	0.058	0.94	0.82–1.08	0.119	0.91	0.76–1.08	0.078	0.87	0.74–1.03	0.105
Ethnicity																	
Caucasian	11	0.98	0.87–1.11	0.031	9	0.87	0.66–1.14	0.069	0.98	0.85–1.12	0.422	0.95	0.83–1.07	0.255	0.88	0.69–1.13	0.069
Asian	1	1.20	0.86–1.67	-	1	1.59	0.73–3.44	-	1.31	0.73–2.35	-	1.36	0.77–2.41	-	1.31	0.69–2.48	-
African	1	0.73	0.51–1.03	-	1	0.50	0.24–1.05	-	0.43	0.23–0.82	-	0.45	0.25–0.83	-	0.90	0.50–1.59	-
End point																	
CHD	9	1.01	0.87–1.17	0.050	7	0.91	0.64–1.29	0.065	0.99	0.82–1.19	0.150	0.96	0.75–1.22	0.093	0.93	0.76–1.15	0.183
MI	4	0.91	0.75–1.11	0.077	4	0.82	0.53–1.26	0.078	0.93	0.78–1.12	0.137	0.90	0.76–1.07	0.109	0.87	0.58–1.31	0.075
Source of controls																	
HB	7	0.95	0.84–1.06	0.156	7	0.88	0.70–1.11	0.123	0.93	0.78–1.12	0.184	0.93	0.78–1.10	0.152	0.94	0.76–1.15	0.203
PB	6	1.02	0.83–1.24	0.013	4	0.89	0.52–1.53	0.030	0.98	0.73–1.31	0.100	0.95	0.70–1.30	0.049	0.88	0.57–1.31	0.070
Sample size																	
Small	6	0.92	0.80–1.06	0.249	5	0.82	0.59–1.15	0.192	0.81	0.56–1.17	0.073	0.82	0.57–1.18	0.057	0.95	0.72–1.27	0.679
Large	7	1.01	0.86–1.19	0.011	6	0.89	0.63–1.27	0.024	1.02	0.88–1.19	0.517	0.98	0.85–1.13	0.264	0.88	0.63–1.22	0.020

*Notes* OR, odds ratio; CI, confidence interval; *P*_H,_ *P* value based on Q test for between-study heterogeneity; HWE =  Hardy–Weinberg equilibrium;

CHD = coronary artery disease; ACS = acute coronary syndrome; MI = myocardial infarction; PB, Population-based; HB, Hospital-based.

**Figure 3:**
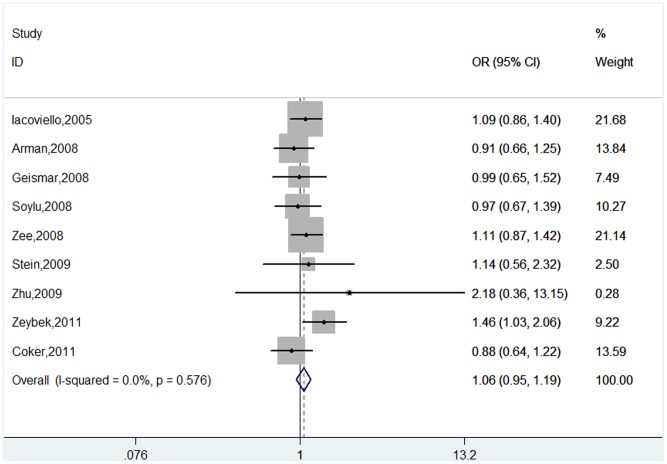
Meta-analysis of the association between *IL-1B +3954* polymorphism and CHD risk (T vs. C).

For *IL-1B+3954* polymorphism, six studies comprised 1,828 cases and 1,818 controls were identified. The overall result suggested no statistically significant association of this polymorphism with CHD susceptibility (T vs. C: OR = 1.06, 95%CI 0.95–1.19; TT vs. CC : OR = 1.18, 95%CI 0.87–1.58; TC vs. CC: OR = 1.12, 95%CI 0.96–1.30; TT+TC vs. CC: OR = 1.13, 95%CI 0.98–1.30; TT vs. TC+CC: OR = 1.12, 95%CI 0.84–1.50) (Table 3, Figure 3). In the subgroup analysis by ethnicity, cardiovascular end point, source of controls and sample size, no significant association was observed in each subgroup under all genetic models (Table 3).

**Table 3:** Summary ORs and 95%CIs of the association between *IL-1B +3954* polymorphism and CHD risk.

Contrasts	No. of studies	T vs. C			No. of studies	TT vs. CC			TC vs. CC			TT+TC vs. CC			TT vs. TC+CC		
		OR	95% CI	*P*_H_		OR	95% CI	*P*_H_	OR	95% CI	*P*_H_	OR	95% CI	*P*_H_	OR	95% CI	*P*_H_
All	9	1.06	0.95–1.19	0.576	8	1.18	0.87–1.58	0.910	1.12	0.96–1.30	0.678	1.13	0.98–1.30	0.592	1.12	0.84–1.50	0.962
Studies in HWE	8	1.02	0.90–1.15	0.892	7	1.09	0.78–1.51	0.969	1.08	0.93–1.27	0.783	1.09	0.93–1.26	0.781	1.06	0.77–1.46	0.981
Ethnicity																	
Caucasian	8	1.06	0.94–1.18	0.537	7	1.18	0.87–1.58	0.910	1.11	0.96–1.29	0.635	1.12	0.97–1.29	0.540	1.12	0.84–1.50	0.962
Asian	1	2.18	0.36–13.15	-	1	-	-	-	2.20	0.36–13.39	-	2.20	0.36–13.39	-	-	-	-
End point																	
CHD	4	0.96	0.78–1.17	0.817	4	0.92	0.54–1.58	0.949	0.96	0.73–1.25	0.655	0.95	0.74–1.23	0.725	0.94	0.55–1.59	0.876
MI	5	1.11	0.97–1.27	0.354	4	1.31	0.92–1.87	0.836	1.20	1.00–1.43	0.728	1.21	1.03–1.44	0.614	1.21	0.86–1.72	0.907
Source of controls																	
HB	6	0.95	0.80–1.12	0.923	4	1.04	0.67–1.63	0.857	1.04	0.83–1.30	0.568	1.04	0.84–1.29	0.570	1.02	0.66–1.59	0.895
PB	3	1.16	1.00–1.36	0.357	4	1.29	0.87–1.93	0.656	1.18	0.97–1.44	0.550	1.20	0.99–1.45	0.430	1.21	0.82–1.79	0.760
Sample size																	
Small	5	1.15	0.94–1.41	0.456	4	1.19	0.73–1.95	0.411	1.21	0.91–1.60	0.574	1.21	0.92–1.57	0.422	1.10	0.68–1.79	0.520
Large	4	1.02	0.89–1.17	0.563	4	1.17	0.80–1.70	0.956	1.09	0.91–1.29	0.479	1.10	0.93–1.30	0.494	1.13	0.78–1.63	0.986

*Notes* OR, odds ratio; CI, confidence interval; *P*_H,_ *P* value based on Q test for between-study heterogeneity; HWE =  Hardy–Weinberg equilibrium;

CHD = coronary artery disease; ACS = acute coronary syndrome; MI = myocardial infarction; PB, Population-based; HB, Hospital-based.

**Figure 4:**
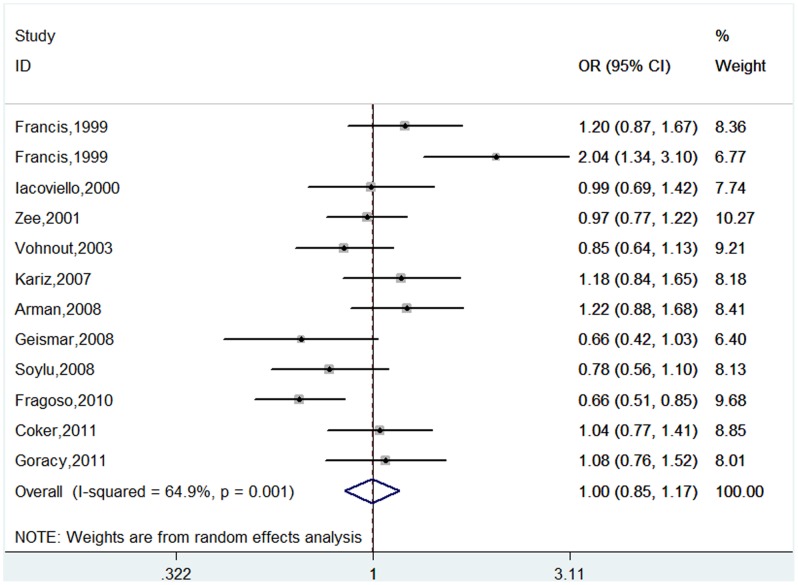
Meta-analysis of the association between *IL-1RN VNTR* polymorphism and CHD risk (*2 vs. L).

For *IL-1RN VNTR* polymorphism, A total of 2,987 cases and 2,208 controls were identified. The overall result suggested no statistically significant association of this polymorphism with CHD risk (*2 vs. L: OR = 1.00, 95%CI 0.85–1.17; *2/*2 vs. L/L: OR = 0.86, 95%CI 0.63–1.18; *2/L vs. L/L: OR = 0.93, 95%CI 0.81–1.07; *2/*2 + *2/L vs. L/L: OR = 0.92, 95%CI 0.81–1.04; *2/*2 vs. *2/L +*2/L: OR = 0.88, 95%CI 0.81–1.04) (Table 4, Figure 4). In the further subgroup analyses based on ethnicity, there was no statistically significant association in all genetic models except for the association in Mixed population under multiplicative, co-dominant and dominant models (Table 4). However the positive association was not reliable because it derived from one study. Subgroup analyses based on cardiovascular end point showed that the effect sizes of *IL-1RN VNTR* with ACS risk were statistically significant under multiplicative, co-dominant and dominant models (Table 4), which also should be interpreted with caution since only one study was included under these three genetic models.

**Table 4:** Summary ORs and 95%CIs of the association between *IL-1RN VNTR* polymorphism and CHD risk.

Contrasts	No. of studies	*2 vs. L			No. of studies	*2/*2 vs. L/L			*2/L vs. L/L			*2/*2 + *2/L vs. L/L			*2/*2 vs. *2/L +*2/L		
		OR	95% CI	*P*_H_		OR	95% CI	*P*_H_	OR	95% CI	*P*_H_	OR	95% CI	*P*_H_	OR	95% CI	*P*_H_
All	12	1.00	0.85–1.17	0.001	9	0.86	0.63–1.18	0.071	0.93	0.81–1.07	0.476	0.92	0.81–1.04	0.194	0.88	0.71–1.10	0.132
Studies in HWE	8	1.12	0.94–1.32	0.043	5	1.06	0.78–1.46	0.215	1.00	0.84–1.20	0.950	1.02	0.86–1.21	0.727	1.06	0.78–1.44	0.246
Ethnicity																	
Caucasian	11	1.04	0.90–1.20	0.020	8	0.94	0.73–1.20	0.127	1.00	0.86–1.16	0.996	0.99	0.86–1.14	0.875	0.94	0.74–1.19	0.132
Mixed	1	0.66	0.51–0.85	-	1	0.5	0.33–0.94	-	0.59	0.41–0.86	-	0.58	0.41–0.82	-	0.68	0.41–1.13	-
End point																	
CHD	7	1.05	0.82–1.33	0.003	5	0.79	0.47–1.33	0.087	1.00	0.82–1.23	0.927	0.96	0.80–1.16	0.602	0.81	0.58–1.13	0.101
MI	4	1.03	0.89–1.19	0.811	3	1.09	0.76–1.56	0.411	1.00	0.80–1.24	0.970	1.02	0.83–1.25	0.905	1.10	0.78–1.55	0.386
ACS	2	0.79	0.53–1.18	0.07	1	0.55	0.33–0.94	-	0.59	0.41–0.86	0.242	-	0.41–0.82	-	0.68	0.41–1.13	-
Source of controls																	
HB	7	0.97	0.86–1.10	0.176	6	0.88	0.66–1.19	0.108	1.00	0.83–1.20	0.968	0.97	0.82–1.15	0.735	0.89	0.67–1.18	0.116
PB	5	1.06	0.77–1.47	0.000	3	0.87	0.49–1.55	0.071	0.85	0.59–1.21	0.063	0.86	0.58–1.15	0.025	0.88	0.63–1.22	0.165
Sample size																	
Small	6	1.05	0.79–1.39	0.004	4	0.82	0.42–1.58	0.065	0.97	0.76–1.23	0.886	0.94	0.75–1.18	0.505	0.83	0.45–1.55	0.076
Large	6	0.95	0.79–1.15	0.024	5	0.85	0.65–1.11	0.124	0.92	0.78–1.08	0.146	0.91	0.72–1.15	0.068	0.89	0.69–1.16	0.231

*Notes* OR, odds ratio; CI, confidence interval; *P*_H,_ *P* value based on Q test for between-study heterogeneity; HWE =  Hardy–Weinberg equilibrium;

CHD = coronary artery disease; ACS = acute coronary syndrome; MI = myocardial infarction; PB, Population-based; HB, Hospital-based.

### Source of heterogeneity

As shown in Table 2, 3, 4, evidence for heterogeneity (*p*<0.10) between studies was found under the multiplicative, co-dominant (TT vs. CC) and dominant genetic models for *IL-1B-511*, under multiplicative and co-dominant (*2/*2 vs. L/L) genetic models for *IL-1RN VNTR* polymorphism, respectively. No evidence for heterogeneity between studies was found for *IL-1B-511* under the co-dominant (TC vs. CC) and recessive genetic models, for *IL-1RN VNTR* under co-dominant (*2/L vs. L/L), dominant and recessive genetic models and for *IL-1B+3954* polymorphism under all the genetic models.

The meta-regression was conducted with the introduction of covariates including ethnicity, publication year, gender, age, sample size, source of controls and cardiovascular end point for the above mentioned polymorphisms. However, no covariate was identified as a potential source of between-study heterogeneity for any comparison.

### Sensitivity analysis

Sensitivity analysis was performed by excluding one study at a time (data not shown) and the non-association results did not substantially alter.

### Potential publication bias

Using Egger's test, no publication bias could be detected for studies published on *IL-1B-511* polymorphism (T vs. C: *p* = 0.388; TT vs. CC: 0.816; TC vs. CC: *p* = 0.592; TT+TC vs. CC: *p* = 0.068; TT vs. TC+CC: *p* = 0.496); IL-1B+3954 polymorphism (T vs. C: *p* = 0.461; TT vs. CC: *p* = 0.231; TC vs. CC: *p* = 0.427; TT+TC vs. CC: *p* = 0.776; TT vs. TC+CC: *p* = 0.691) and *IL-1RN VNTR* polymorphism (*2 vs. L: *p* = 0.295; *2/*2 vs. L/L: *p* = 0.172; *2/L vs. L/L: *p* = 0.152; *2/*2 + *2/L vs. L/L: *p* = 0.106; for *2/*2 vs. *2/L +*2/L: *p* = 0.855).

## Discussion

Cytokines of the IL-1 family were believed to influence the inflammatory response and inflammation-related atherosclerosis, and these in turn lead to CHD and other cardiovascular diseases such as stroke [3], [45], [46]. Because of the effects of cytokines on inflammatory response, a series of studies have focused on the contribution of polymorphisms within the IL-1 cluster genes to the CHD risk. However, results have been contradictory. Given the relatively small sample sizes of the included individual studies for detecting the modest genetic effect, we conducted the present meta-analysis, although there were still the limited power of meta-analysis due to size and heterogeneity of studies/patients.

Our results suggested that there was no significant association between the three polymorphisms (*IL-1B-511*, *IL-1B+3954* and *IL-1RN VNTR*) within the *IL-1* gene cluster and CHD risk. One previous meta-analysis also failed to suggest statistically significant associations of *IL-1B-511* and *IL-1RN VNTR* polymorphisms with stroke risk in the overall population, with ORs and 95%CI of 1.22 (0.85–1.87) for TT vs. CC and 1.22 (0.85–1.75) for RN2/RN2 vs. RN1/RN1 [47]. In addition, the previous meta-analysis by Chen et al in Chinese showed that *IL-1* gene cluster polymorphisms did not seem to affect CHD risk, with ORs and 95%CI of 1.04 (0.93–1.18) for *IL-1B-511* and 1.01 (0.78–1.17) for *IL-1RN VNTR* under multiplicative models [30].

IL-1β, released by macrophages, platelets, and injured endothelium [48], plays a central role in the inflammatory response and its related atherosclerosis. IL-1β may act on atherosclerosis with different biological functions such as stimulating proliferation of vascular smooth muscle cells and endothelial cells [49], [50], increasing expression of adhesional molecule from endothelial cells [50], modificating the endothelium to promote coagulation and thrombosis [51], stimulating the synthesis of fatty acid carrier protein by adipose tissue in vitro [52], promoting the production of some other pro-inflammatory factors such as IL-6, fibrinogen and C-reactive protein [45], [46]. At the same time, IL-1Ra regulates inflammation by functioning as an endogenous inhibitor of IL-1β and competing for IL-1 receptor. Therefore, the balance between IL-1β and IL-1Ra is thought to contribute to the pathogenesis of atherosclerosis [45], [46]. In addition, some evidence has indicated that elevated levels of IL-1 and IL-1Ra mRNA were observed in atherosclerotic arteries compared with normal arteries [53]. In addition, T allele of the *IL-1B-511* polymorphism and 2 allele of the *IL-1RN VNTR* polymorphism have been associated with enhanced IL-1b production [54], [55]. Nevertheless, no significant association was found between *IL-1* gene cluster polymorphisms and cardiovascular diseases by meta-analysis.

Considering that CHD is a multifactorial trait and the impact of the inflammatory cytokine on CHD progress may be modulated by age, gender and some other environmental and genetic factors across different ethnicities, the subgroup analysis based on ethnicity was performed, which showed that *IL-1B-511* polymorphism was only associated with CHD in Africans under co-dominant and dominant models, *IL-1RN VNTR* polymorphism associated in Mixed population under multiplicative, co-dominant and dominant models, respectively. However, the results were not very credible due to just one study included in the Africans and Mixed population separately.

Furthermore, the subgroup analysis indicated the positive association of *IL-1RN VNTR* polymorphism with ACS but not with other cardiovascular end point. However, what also needs to be pointed out is that the significant association derived from only one study and thus the result should be interpreted with caution because of the relatively small sample size or multiple testing driving false positive findings.

There are several limitations in the meta-analysis. First, our analysis was primarily based on unadjusted effect estimates and therefore the potential covariates including age, gender and environmental factors such as smoking and levels of HDL-cholesterol, which might influence the effect estimates, were not controlled for. Second, despite of evidence of between-study heterogeneity in some comparisons in our meta-analysis, none of the covariates including ethnicity, publication year, gender, age, sample size, source of controls and cardiovascular end point was identified as a potential source of heterogeneity between studies by meta-regression. Therefore, other unknown confounding factors may help explain the between-study heterogeneity. Third, the possibility of a false negative remains due to the small size of the studies even when combined. Thus, further studies with larger sample size are required to investigate the associations. Fourth, as none of the studies included in this meta-analysis considered the effect of gene-gene/environment interactions involved in the pathogenesis of CHD, this issue could not addressed in our meta-analysis. Fifth, as is known, haplotype analysis might bring out bigger net effects. However, most studies, except for the studies by Zee et al [13]and Fragoso et al [25], did not perform haplotype analyses, which impeded our further analysis. Sixth, it is conceivable, that patients with a higher inflammatory status and a polymorphism in IL1B or IL1RA have stronger association to coronary heart disease than patients without inflammation. Analysis of hsCRP in a subgroup could help to answer this question. Three studies (the study by Iacoviello et al [16], Soylu et al [21] and Coker et al [27]) provided the data about hsCRP. However, only the study by Iacoviello et al [16] provided the hsCRP-adjusted OR with 95% CI; the other two studies by Soylu et al [21] and Coker et al [27] only provided the hsCRP levels between cases and controls. Therefore, subgroup analysis of the effect of hsCRP on variant-CHD association can not yet been conducted so far.

In summary, our meta-analyses suggested that *IL-1* gene cluster polymorphisms were not associated with CHD risk. More in depth researches considering gene-environment interactions and haplotype information should be conducted to further investigate these associations between *IL-1* gene cluster polymorphisms and CHD risk.
